# AI Integration in Indonesian Education: Trends, Outcomes, and Challenges - A Comprehensive Review

**DOI:** 10.12688/f1000research.188536.3

**Published:** 2026-10-26

**Authors:** Aulia Riski, Reinhard Leonardo Paais, Aan Komariah, Novebri Novebri, Lany Pranita Alang, Winda Verawati Ataupah, Zeffa Yurihana, Maria Vatmawaty Bety, Denny Ramadhan, Ade Syarifudin, Linda Dwiyanti, Teuku Yasser

**Affiliations:** 1 Educational Administration, Universitas Pendidikan Indonesia, Bandung, West Java, 140154, Indonesia; 2 Islamic Educational Management, Sekolah Tinggi Agama Islam Negeri Mandailing Natal, Mandailing Natal, North Sumatera, 22976, Indonesia

**Keywords:** Artificial Intelligence in Education, AI Implementation, Adaptive Learning, Systematic Literature Review, Indonesian Education, Quality Education (SDGs 4)

## Abstract

**Background:**

Global education trends show the rapid growth of Artificial Intelligence (AI) as one of the leading technologies worldwide, a shift increasingly reflected in Indonesian education. This study aims to provide a Systematic Literature Review (SLR) by mapping the research landscape, its representation in practice and policy, its impact on learning quality, and the barriers to AI integration in Indonesian education.

**Method:**

Using the PRISMA protocol, 34 peer-reviewed articles indexed in the Scopus and Google Schoolar databases (2020–2026) were systematically selected and thematically analyzed across four main themes.

**Results:**

The findings indicate a rapid increase in publication productivity, particularly following the worldwide expansion of generative AI technologies after 2022. However, this growth remains unevenly distributed, concentrated in higher education institutions and urban centers on the island of Java, with a marked absence in primary and secondary education levels and in regions outside Java. The majority of AI-driven implementations consist of generative chatbots, adaptive learning platforms, and intelligent tutoring systems, alongside emerging applications such as learning analytics and AI-powered instructional media. Several studies included in this review reported that AI has contributed to measurable improvements in student outcomes including learning achievement, motivation, engagement, and teaching efficiency in well-implemented AI environments. Nonetheless, its adoption also poses significant risks, including a decline in students’ critical thinking skills, threats to academic integrity, and the absence of comprehensive data privacy policies and ethical standards governing AI use in education.

**Conclusion:**

These findings underscore the urgent need for a responsible AI implementation framework in Indonesia one that incorporates standardized AI literacy competencies for teachers, locally contextualized ethical policies, and a functional national roadmap for AI-enabled educational transformation.

## Introduction

Over the past ten years, the education sector has seen a dramatic acceleration of digital transformation, with artificial intelligence (AI) emerging as one of the most disruptive technologies, transforming traditional learning paradigms into more efficient, personalized, and adaptable systems. The incorporation of AI into the educational ecosystem is no longer just an extra innovation in the period of the Industrial Revolution 4.0, which is currently moving toward Society 5.0, but rather a strategic requirement to equip the next generation to compete in a world that is becoming more and more competitive. Artificial intelligence (AI) technologies, such as computer vision, machine learning, natural language processing, and intelligent tutoring systems ( Athallah, 2025; Lin et al., 2023; Rahman, 2025; Sucianingtyas et al., 2025; Novebri et al., 2026), offer enormous potential to automate administrative tasks, provide real-time feedback, and identify learning patterns that improve pedagogical effectiveness. However, integrating AI into education, especially in developing nations like Indonesia, poses a number of difficult issues that call for thorough and methodical research. These issues include technology infrastructure, human resource preparedness, regulatory laws, and sociocultural aspects.

With a population of over 270 million and an educational system that serves almost 50 million students at different levels, Indonesia is the largest archipelagic nation in the world. As such, it has particular difficulties in guaranteeing fair education quality. National education stakeholders have long been concerned about structural issues such as gaps in digital competencies among educators, limited access to contemporary learning technologies, and disparities in education quality between urban and rural areas ( Adekamisti et al., 2025; Idin et al., 2024; Olanrewaju et al., 2021; Saputra, 2025). However, Indonesia enjoys a demographic dividend because most of its people are of working age, tech-savvy, and innovative, making them valuable social capital for AI-driven educational change. The Merdeka Mengajar Platform, the Sekolah Penggerak program, and other digital learning initiatives are just a few of the policies and programs that the Indonesian government, through the Ministry of Education, Culture, Research, and Technology, has introduced to promote education digitalization. These circumstances provide a timely impetus to investigate how AI might be successfully applied in the Indonesian educational setting while taking into account regional traits, particular requirements, and current obstacles.

Numerous studies showing favorable effects on student engagement, learning personalization, administrative efficiency, and analytical capabilities in educational decision-making have contributed to the rapid global expansion of research on AI implementation in education ( Ellikkal & Rajamohan, 2025; Rochmawati et al., 2023; Sabariah et al., 2024; Sajja et al., 2025). While AI-based learning analytics systems can identify at-risk students early and enable proactive intervention ( Gupta et al., 2022; Ravichandran et al., 2023), intelligent tutoring systems have been shown to offer personalized guidance tailored to each student’s learning pace and style ( Akyuz, 2020; Bradáč et al., 2022). In order to create more relevant and successful learning experiences, adaptive learning platforms use machine learning algorithms to modify learning pathways and material difficulty based on student performance and preferences ( Al Fadillah & Akbar, 2024; Gligorea et al., 2023). In order to facilitate more successful self-directed learning, educational chatbots and virtual teaching assistants can provide 24/7 learning support, respond to student questions, and give immediate feedback. The need for contextualized research that takes into account local realities is highlighted by the fact that the majority of these studies have been carried out in developed nations with sophisticated technological infrastructure, standardized educational systems, and digital ecosystems that are fundamentally different from Indonesia.

Although there is a lot of excitement about how AI can change Indonesia’s educational system, there are still several obstacles that need to be taken into consideration. Adoption of AI technologies, which require reliable internet access and sufficient computing devices, is still fundamentally hampered by a lack of technological infrastructure, particularly in isolated areas of Eastern Indonesia ( Alabdali et al., 2023; Sinaga & Harahap, 2025). Since successful AI adoption depends not only on technological competence but also on instructors’ capacity to pedagogically incorporate these technologies into learning processes, the digital literacy gap among educators is a significant challenge ( Entriza & Puspitasari, 2025; A. S. George, 2023; Tedre et al., 2021). High upfront expenses for software license, infrastructure acquisition, and human resource training sometimes serve as obstacles for educational institutions, especially in areas with limited funding ( Do et al., 2025; Renz & Hilbig, 2020; Shang et al., 2023; Winarsih, 2025). Concerns about algorithmic bias that could worsen educational inequality, the privacy and security of student data, and the possible dehumanization of learning processes that ought to prioritize human interaction between teachers and students are also present ( Kaul, 2026; Lata, 2024; Marifahtullah & Faizi, 2024; Sholihah & Abi Aufa, 2025). In order to find best practices, lessons learned, and implementation techniques that are relevant for Indonesia’s setting, these difficulties require thorough examination.

Systematic Literature Review (SLR) is one of the accepted methodologies to thoroughly, impartially, and methodically map the research landscape on AI application in Indonesian education. Unlike conventional literature reviews, which tend to be narrative and selective, SLR employs transparent and replicable procedures to gather, evaluate, and synthesize all relevant data for particular research topics, minimizing bias while enhancing the validity of conclusions. SLR can identify research trends, gaps in knowledge, context of implementation, reasons for success and failure, and useful advice for different education stakeholders. A systematic approach of this kind also enables us to identify Indonesia-specific patterns of AI implementation, distinguish local innovation from the adaptation of global technology, and explore the impact of sociocultural, policy, and infrastructure factors on the trajectory of AI integration in education. Therefore, SLR provides a state-of-the-art research summary and an evidence-based foundation for the policy development and the practical application of AI in Indonesian education.

The application of AI on education in Indonesia has several significant issues that need to be investigated in detail through a systematic literature review. Firstly, it is necessary to examine the trends and developments in research on AI implementation in learning in Indonesia, including patterns of publication, research contexts, educational levels, and emerging areas of investigation. Second, attention needs to be given to the forms, models, and strategies of AI implementation in the learning process, including the types of AI technologies adopted and how they are integrated into teaching and learning practices. Third, it is important to examine the impact of AI implementation on the quality of learning in Indonesia, particularly in relation to learning outcomes, student engagement, motivation, learning effectiveness, and other dimensions of learning quality reported in the literature. Fourth, the review needs to identify the challenges, limitations, and opportunities associated with the development of AI implementation in learning in Indonesia, including technological, pedagogical, organizational, sociocultural, ethical, and regulatory considerations where these emerge from the reviewed studies. These questions guide the systematic review process to generate comprehensive and useful knowledge synthesis.

This systematic literature review’s main goals are to map Indonesia’s research landscape on AI implementation in education, spot trends, patterns, and gaps in the literature, and offer evidence-based insights to guide future research, practice, and policy. The specific objectives of this study are to: (1) identify and classify different AI applications and technologies used in Indonesian education; (2) examine factors that help and hinder AI implementation in Indonesian education; (3) assess the impact and efficacy of AI implementation on various learning outcomes dimensions; (4) investigate research methodologies used in studies of AI implementation in Indonesian education; (5) identify best practices, lessons learned, and recommendations for effective and sustainable AI implementation in Indonesia; and (6) develop a future research agenda to strengthen the evidence base for AI-driven educational transformation in Indonesia. By accomplishing these goals, this research is anticipated to make a substantial contribution to the advancement of theory and practice in the application of AI in education, especially in emerging nations with distinctive features like Indonesia.

Although a growing number of reviews have examined the educational applications of artificial intelligence, most of them adopt a global perspective or focus on a single dimension of AI use, such as generative AI in higher education, the effectiveness of a particular tool, or ethical concerns in isolation. Reviews that address the Indonesian context remain limited. Those that exist tend to describe the general potential of AI rather than systematically linking research trends, implementation approaches, reported learning outcomes, and adoption challenges within one analytical framework. This gap matters because Indonesia’s educational system has distinctive characteristics, including wide disparities in digital infrastructure between Java and other regions, a large primary and secondary education sector, and an AI policy framework that is still emerging. These conditions may shape how AI is researched and implemented in ways that global syntheses cannot capture. This review therefore makes four contributions. First, it integrates research trends, implementation approaches, educational outcomes, and challenges of AI integration into a single systematic synthesis. Second, it maps how the existing evidence is distributed across educational levels and geographical regions in Indonesia. Third, it identifies methodological and contextual gaps in the evidence base, particularly the scarcity of experimental designs and the underrepresentation of primary and secondary education and of regions outside Java. Fourth, it translates these findings into implications for Indonesian education policy, including AI literacy for teachers, ethical and data privacy governance, and a national roadmap for AI-enabled educational transformation.

The systematic literature review has several contributions that are relevant for various stakeholders in Indonesia’s education ecosystem. This assessment gives useful advice on established AI uses; appropriate ways to put them into practice and anticipating possible problems to be encountered when introducing technology to teachers such as teachers, tutors and school heads. The comprehensive evidence synthesis can support national and regional officials in developing policies, allocating resources, and planning programs that promote fair and sustainable AI integration into the country’s education system. Awareness of knowledge gaps and future research priorities can help direct researchers to impactful and contextually relevant research. Understanding the needs, challenges, and preferences of Indonesian users can assist tech companies and the edtech industry in developing AI products and solutions that are better adapted to the local context. More broadly, this study contributes to the worldwide conversation on AI in education by providing perspectives from a developing country, contributing to the literature that often neglects contexts and providing insights into how global technologies are adapted to specific local contexts with their challenges and opportunities.

## Methodology

The aim of this project is to extensively map, analyze and integrate scientific data on the application of artificial intelligence (AI) in education in Indonesia. In particular, it discusses trends and developments of research, models and types of AI application, their impacts on the quality of learning, and prospects and challenges of their development in the context of Indonesian education. This approach attempts to create a rich, factual understanding of the strategic role of AI in Indonesian education.

The Systematic Literature Review (SLR) method applied in this study is based on the Preferred Reporting Items for Systematic Literature Reviews and Meta-Analyses (PRISMA) framework. The PRISMA framework ensures the clear, systematic and reproducible manner of literature search, selection, evaluation and synthesis. The main steps of this study are: article selection. During the article screening and selection phase, search results were first reviewed by several co-authors based on pre-established inclusion and exclusion criteria; subsequently, a professor in the field of education conducted an expert assessment to validate the suitability of the articles. Any discrepancies in assessment were resolved through discussion and consensus before a final decision was made to include the articles in the review. The next stage involved a comprehensive review of eligibility, followed by the analysis and synthesis of findings relevant to the research focus. Ultimately, this entire process yielded 34 articles suitable for review.

This study is designed to address four main research questions: (RQ1) What are the trends and developments in research on the implementation of artificial intelligence in learning in Indonesia?; (RQ2) What forms, models, and strategies of artificial intelligence implementation are used in the learning process in Indonesia?; (RQ3) What is the impact of artificial intelligence implementation on the quality of learning in Indonesia?; and (RQ4) What are the challenges, limitations, and opportunities for developing artificial intelligence implementation in learning in Indonesia? These four questions serve as the conceptual foundation for data coding, thematic categorization, and interpretation of the research findings.

The articles search was conducted on 8 February 2026 using Scopus and Google Scholar. The search targeted publications published between 2020 and 2026 that addressed artificial intelligence in the context of Indonesian education. The retrieved records were subsequently assessed against the predefined inclusion and exclusion criteria. Furthermore, this time frame enables a more contextual analysis of the impact of learning techniques, educational regulations, and technological changes on the adoption of AI in Indonesia in the contemporary age.

Figure 1 shows how the literature for this study was chosen. The four primary steps of the PRISMA (Preferred Reporting Items for Systematic Reviews and Meta-Analyses) protocol identification, screening, eligibility assessment, and inclusion were followed in the data gathering process. During the identification phase, a total of 773 articles were obtained from two databases: Google Schoolar (n = 643) and Scopus (n = 130). There were 653 articles left for screening after a number of articles were eliminated before the screening step, including 52 duplicate records, 36 items that automated tools marked as ineligible, and 32 articles that were omitted for various reasons.

**Figure 1.**
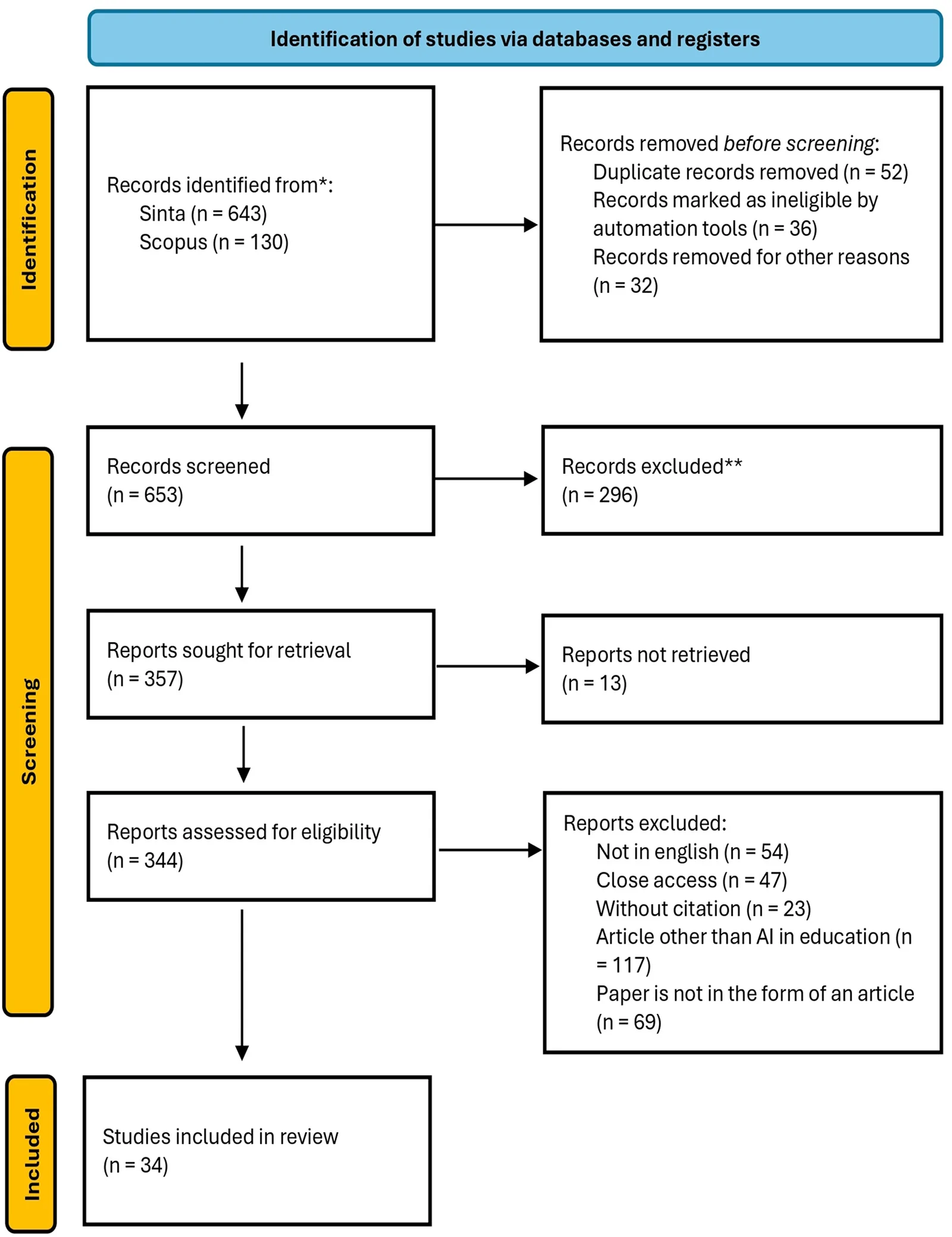
Review Protocol Using PRISMA.

357 articles were searched for full-text retrieval after 296 of the 653 articles were eliminated based on title and abstract during the screening phase. Of these, 344 articles were left for full-text eligibility evaluation after 13 could not be obtained. Articles that did not fit the inclusion criteria were further excluded at the eligibility stage. Articles published in languages other than English or Indonesian (n = 54) were excluded during the eligibility assessment. (n = 54), articles with restricted access (n = 47), articles without citations (n = 23), articles unrelated to AI in education (n = 117), and publications that were not in the form of scientific articles (n = 69). The authors excluded articles without citations and those published in closed-access formats to prioritize studies with demonstrable scholarly visibility and accessible full-text content for comprehensive examination and consistent thematic analysis. 34 papers were utilized as the main data sources for the synthesis in this systematic literature review after completing all phases of this stringent selection process and meeting all inclusion criteria.

Table 1 shows the methodically specified inclusion and exclusion criteria that were used to pick the publications for this investigation. The included papers are journal articles published between 2020 and 2026 that are open-access, authored in either English or Indonesian, and focus on artificial intelligence (AI) in educational institutions. They are sourced from the Scopus and Google Schoolar databases. On the other hand, articles that were not written in English, had incomplete or restricted access, lacked citations, or addressed subjects unrelated to artificial intelligence (AI) in education were disqualified. 34 publications that satisfied the requirements for additional analysis were found using the search terms Artificial Intelligence, Machine Learning, Learning, Teaching, and Artificial Intelligence in Education Indonesia.

**Table 1.** Inclusion And Exclusion Criteria.

Search Keyword	Artificial Intelligence, Generative Artificial Intelligence, Generative AI, Machine Learning, Intelligent Tutoring System, AI in Education	
**Inclusion Criteria**	Time line	Publication year: 2020 to 2025
	Document type	Article Journal (research article and conference paper)
	Language	English and Indonesia
	Source	Scopus and Google Schoolar
	Geographical areas	All around the world
	Subject area	Social science and education
**Exclusion Criteria**	Time line	<2020
	Document type	Book chapter, review, note, editorial, book, letter, retracted, short survey, thesis
	Language	Languages other than English and Indonesia
	Source	Except Scopus and Google Schoolar
	Geographical areas	
	Subject area	Besides social sciences, education
**Search String**	Scopus	(TITLE-ABS-KEY((“artificial intelligence” OR AI OR “generative artificial intelligence” OR “generative AI” OR ChatGPT OR “large language model*” OR “machine learning” OR “deep learning” OR “learning analytics” OR “intelligent tutoring system*” OR “adaptive learning” OR chatbot*) AND (education OR learning OR teaching OR curriculum OR classroom OR school* OR university* OR college* OR “higher education” OR “primary education” OR “secondary education”) AND (Indonesia OR Indonesian))) AND PUBYEAR >2019 AND PUBYEAR <2027–130 documents found
	Google Scholar	(“artificial intelligence” OR AI OR “generative artificial intelligence” OR “generative AI” OR ChatGPT OR “large language model” OR “machine learning” OR “deep learning” OR “learning analytics” OR “intelligent tutoring system” OR “adaptive learning” OR chatbot) AND (education OR learning OR teaching OR curriculum OR classroom OR school OR university OR college OR “higher education” OR “primary education” OR “secondary education”) AND (Indonesia OR Indonesian)

## Result and Discussion

### Result

The key conclusions from the systematic literature review of 34 chosen scientific publications are summarized in this section ( Riski, 2026). The forms, models, and strategies of artificial intelligence implementation used in Indonesian learning processes; the effect of AI implementation on Indonesian learning quality; and the obstacles, constraints, and prospects for the advancement of AI implementation in Indonesian learning were the four primary research focuses into which the analysis was conducted thematically.

The goal of presenting the results in tabular form is to offer a methodical, comparative, and structured mapping of research patterns across different methodological and geographic contexts. This method provides a solid analytical basis for more discussion in the following section by making it possible to identify dominating patterns, thematic variations, and correlations among pertinent variables. Table 2 displays an analysis of the distribution of articles:

**Table 2.** Analysis of Article Distribution.

Code	Author	Title	References
A1	Dini Noor Arini, Fahmi Hidayat, Atiek Winarti, and Elsa Rosalina	Artificial intelligence (AI)-based mobile learning in ELT for EFL learners: The implementation and learners’ attitudes	( Arini et al., 2022)
A2	Renika Hasibuan, Ida Bagus Made Wisnu Parta, Husna Imro’athush Sholihah, Antono Damayanto, Farihatun	Transformation of Indonesian Language Learning with Artificial Intelligence Applications: The Era of the Independent Curriculum for Learning in Universities in Indonesia	( Hasibuan et al., 2023)
A3	Kaharuddin, Djuwairiah Ahmad, Mardiana, Ismail Latif, Burhanuddin Arafah, Ray Suryadi	Defining the Role of Artificial Intelligence in Improving English Writing Skills Among Indonesian Students	( Ahmad et al., 2024)
A4	Kukuh Andri Aka, Punaji Setyosari, Endang Purwaningsih, Mardhatillah	Enhancing Primary Education in Indonesia: Integrated Learning Policies And Artificial Intelligence Utilization	( Aka et al., 2024)
A5	Julianus Labobar, Yakob Godlif Malatuny	Artificial Intelligence: Challenges in Civic Education Learning	( Labobar & Malatuny, 2024)
A6	Neka Zulwiddi	Implementation of Artificial Intelligence in Fiqh Learning to Improve Student Activeness	( Zulwiddi, 2023)
A7	Putu Trisna Hady Permana, Ni Luh Putu Ning Septyarini Putri Astawa	Artificial Intelligence in the Development of English Language Learning Media	( Permana & Putri, 2020)
A8	Agnes Deta Waluyaningtyas, Saryanto, Rejokirono	Utilization of Artificial Intelligence in Strengthening the Pancasila Student Profile Project Integrated with STEM	( Waluyaningtyas et al., 2024)
A9	A.Aviv Mahmudi, Richa Fionasari, Budi Mardikawati, Loso Judijanto	Integration of Artificial Intelligence Technology in Distance Learning in Higher Education	( Mahmudi, 2023)
A10	Vivi Puspita, Shella Marcelina, Silfi Melindawati	Training on the Use of Artificial Intelligence in Developing Learning Modules for Elementary School Teachers	( Puspita et al., 2023)
A11	Haris Setyawan, Dwijoko Purbohadi	Experimenting with AI-based mobile applications to improve student engagement in ornamental plant learning in rural Indonesian schools	( Setyawan & Purbohadi, 2025)
A12	Adiyono Adiyono, Tono Suwartono, Sri Nurhayati, Fahmy Ferdian Dalimarta, Okto Wijayanti	Impact of Artificial Intelligence on Student Reliance for Exam Answers: A Case Study in IRCT Indonesia	( Adiyono et al., 2025)
A13	Nafri Yanti, Arono, Fina Hiasa, Febi Junaidi, Noermanzah, Rio Kurniawan	Indonesian Teachers’ Roles in Designing and Utilizing AI-Powered Animated Videos: A Case Study on Classroom Practices and Character Development	( Utilizing, 2025)
A14	Idha Novianti, Elang Krisnadi, Thesa Kandaga, Ranak Lince, Husnaeni, Nurmawati, Sudirman Sudirman	Personalization Imperative: Unpacking Student-AI Relationships Through a Mixed-Methods Lens in Indonesian Higher Education	( Novianti et al., 2026)
A15	Iman Santoso, Rani Supriyadi, Widya Utami	The Role of Artificial Intelligence in Indonesia’s Digital Transformation: Challenges and Opportunities in 2024	( Santoso et al., 2024)
A16	Fanni Yunita, Gunawan	Artificial Intelligence in Mathematics Learning: A Challenge and Opportunity	( Yunita & Gunawan, 2025)
A17	Adinda Arly, Nanda Dwi, Rea Andini	Implementation of Artificial Intelligence in the Learning Process of Communication Science Students in Class A	( Arly et al., 2023)
A18	Velda Aurelia Putri, Kadek Carissa Andjani Sotyawardani, Raihan Andre Rafael	The Role of Artificial Intelligence in the Learning Process of Students at Universitas Negeri Surabaya	( Putri et al., 2023)
A19	Nagy, A.S., Tumiwa, J.R., Arie, F.V., & Erdey, L.	An exploratory study of artificial intelligence adoption in higher education	( Nagy et al., 2024)
A20	Ramdania, D.R., Pradana, H., & Fauzi, F.R.	Analysis of the Utilization of Artificial Intelligence Technology in Learning in Indonesia	( Ramdania et al., 2023)
A21	Ninghardjanti, P., Subarno, A., Winarno, & Umam, M.C.	Evaluating the Intention for the Student’s Adoption of Artificial Intelligence for Learning Activities in Education-Based University	( Prameka et al., 2024)
A22	Al Yakin, A., Muthmainnah, M., Cardoso, L., Al Matari, A.S., & Obaid, A.J.	AI-powered Personalization for Learning and Human-Robot Interaction: A Case Study with Pre-Service Teachers from Indonesia	( Al Yakin et al., 2025)
A23	Dinata, R.P., Suryati, N., Jailani, M.K., Keli, Y.P., Rovikasari, M., & Hasymi, M.	Exploring Rural EFL Lecturers’ Perspectives on the Integration of Artificial Intelligence (AI) in Foreign Language Pedagogy	( Dinata et al., 2025)
A24	Syaidina, M.O., Fahrudin, R., & Mutiara, I.A.	Implementation of Ethics of Using Artificial Intelligence in the Education System in Indonesia	( Syaidina et al., 2024)
A25	Raharjo, R. S. & Rohmadi, S. H.	Artificial Intelligence in Indonesian Education: A Critical Review of Ethical Considerations, Implementation Challenges, and Educational Management Perspectives	( Raharjo & Rohmadi, 2025)
A26	Fahmi, M. & Adhimah, S.	The Role of Artificial Intelligence in Arabic Language Learning: Opportunities and Challenges	( Fahmi & Adhimah, 2024)
A27	Utami, S. P. T., Andayani, Winarni, R., & Sumarwati	Utilization of artificial intelligence technology in an academic writing class: How do Indonesian students perceive?	( Utami & Winarni, 2023)
A28	Ninghardjanti, P., Umam, M. C., Subarno, A., Winarno, W., Langgi, N. R., & Widodo, J.	Evaluating the impact of AI on the critical thinking skills among the higher education students by combining the TAM model and critical thinking theory	( Ninghardjanti et al., 2026)
A29	Helmiatin, Hidayat, A., & Kahar, M. R	Investigating the adoption of AI in higher education: a study of public universities in Indonesia	( Helmiatin et al., 2024)
A30	Putri Supriadi, S. R. R., Sulistiyani, & Chusni, M. M.	AI-Based Learning Innovation in Education in the Era of Industry 4.0 and Society 5.0	( Supriadi et al., 2022)
A31	Juwika Afrita	The Role of Artificial Intelligence in Improving the Efficiency and Effectiveness of the Education System	( Afrita, 2023)
A32	Heinrich Rakuasa, Dzaka Ashriel Faris, Muh. Hidayatullah	Transforming Education in the Age of Artificial Intelligence: Challenges and Opportunities in Indonesia, A Literature Review	( Rakuasa et al., 2024)
A33	Md Nurul Islam et al. (8 authors)	Application of artificial intelligence in academic libraries: a bibliometric analysis and knowledge mapping	( Islam et al., 2025)
A34	Yeyen Fitri, Daroe Iswatiningsih	Implementation of Artificial Intelligence in Elementary School Learning: Opportunities and Challenges for Teachers at SDN Madang Musi Rawas	( Fitri & Iswatiningsih, 2025)

### RQ1: What are the trends and developments in research regarding the implementation of artificial intelligence in learning in Indonesia?

The rapid digital transformation of the education sector is reflected in the expanding trend of research on the application of AI in Indonesian education. Several publications that explicitly look at the use of AI in Indonesian education were found based on the findings of the literature selection process carried out through a comprehensive literature review. These articles provide a thorough overview of the research landscape of AI in education in Indonesia by covering a variety of time periods, educational levels, AI technologies used, and topics of study. The results from each study were examined using the chronological, thematic, and technological aspects employed to address RQ1, as shown in the accompanying Table 3.

**Table 3.** Distribution of Articles Based on Publication Patterns, Fields, Levels, and Methods.

Aspect	Category	Count	Notes
**A. Publication Trends (34 articles, 2020–2026)**			
**Year of Publication**	2020–2022	**3 articles**	Early phase; limited research output
	2023–2024	**20 articles**	Significant growth following generative AI emergence
	2025–2026	**11 articles**	Publication peak (47.1% of total)
**B. Education Level**			
**Education Level**	University/Higher Education	**22 articles**	Dominant (64.7%) — AI adoption in higher education
	Junior High School	**5 articles**	14.7%
	Elementary & Senior High School	**4 articles**	11.8% each
	Multi-level	**3 articles**	Cross-level studies
**C. Research Methods**			
**Research Method**	Qualitative/Descriptive	**13 articles**	38.2% — interviews, observations, case studies
	Literature Review/Systematic Review	**8 articles**	23.5% — SLR, bibliometrics, policy review
	Quantitative (survey, SEM, quasi-experiment)	**7 articles**	20.6%
	Mixed Methods	**6 articles**	17.6% — combination of qualitative & quantitative
**D. Research Domain/Focus**			
**Main Topic**	Language Learning (English, Indonesian, Arabic)	**9 articles**	Most frequent topic; EFL, writing, digital media
	AI Adoption & Institutional Readiness	**5 articles**	UTAUT, TAM, SEM-PLS models
	Ethics, Policy & AI Governance	**4 articles**	Academic integrity, SWOT, regulation
	Digital Transformation & Learning Innovation	**4 articles**	Industry 4.0, Society 5.0, smart city
	Mathematics & Natural Sciences	**3 articles**	AI personalization, SLR, PBL
	Others (Library, Administration, etc.)	**9 articles**	Diverse sectors in education
**E. Research Location Distribution**			
**Region**	Java (Jakarta, Yogyakarta, Surabaya, etc.)	**21 articles**	61.8% — concentration of research in Java
	Sulawesi (Manado, Makassar, West Sulawesi)	**4 articles**	11.8%
	Sumatra (Padang, Bukittinggi, Banten)	**4 articles**	11.8%
	Kalimantan, Papua, Bali	**3 articles**	8.8%
	Multi-location/National	**2 articles**	5.9%

Figure 2 shows a fluctuating development trend throughout the observation period. The initial value was 1, then decreased to 0 before gradually increasing to 2. After that, there was a significant spike to 9, although there was a slight decrease to 8 in the following period. The trend increased again and peaked at 12, the highest value during the observation period. However, after reaching this peak, there was a very sharp decline, returning to 2 in the final period. This pattern indicates a phase of strong growth in the middle of the period, followed by a drastic decline at the end, so further analysis is needed to identify the factors influencing this change.

**Figure 2.**
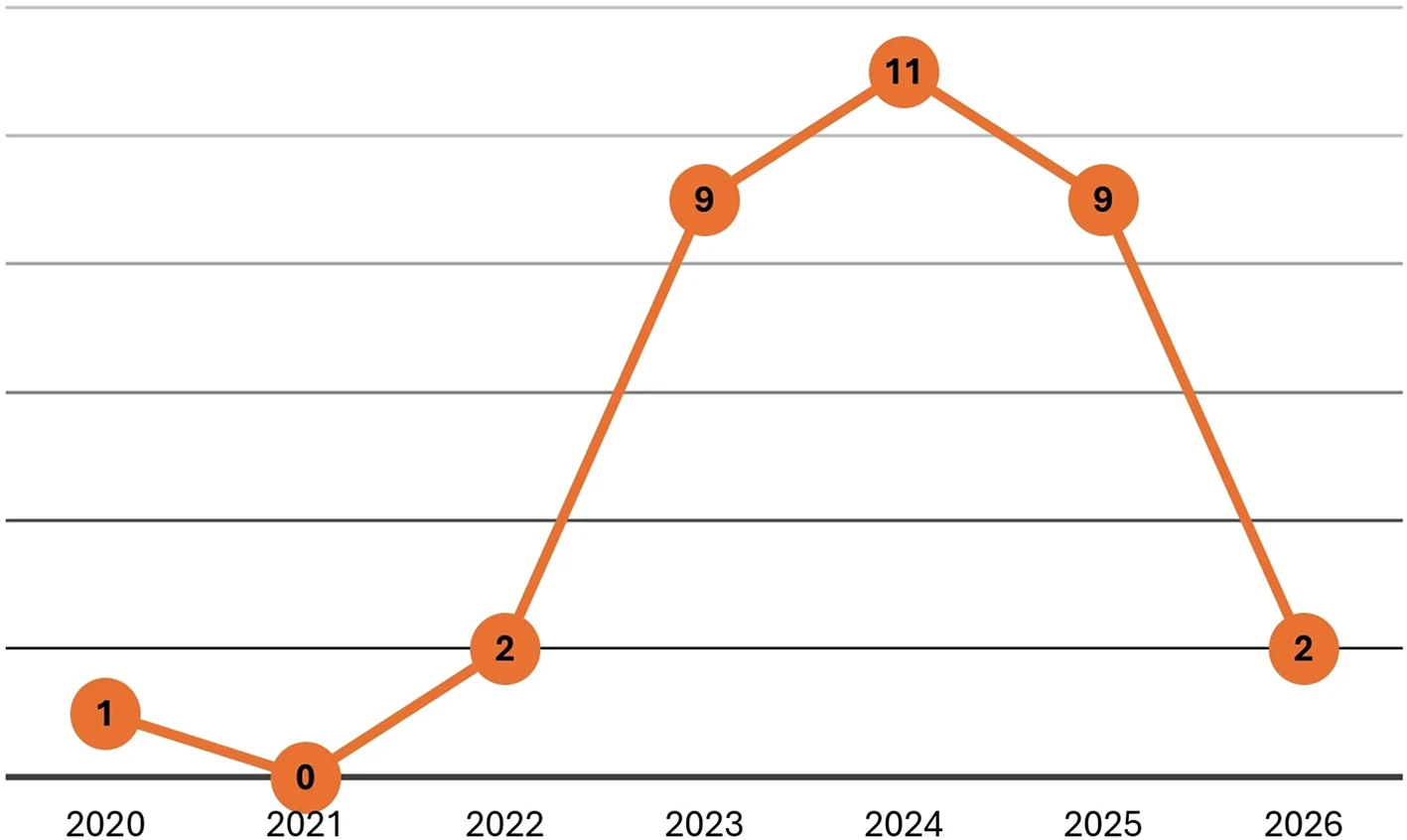
Distribution of articles based on year of publication.

The research trend on the use of artificial intelligence (AI) in Indonesian education has significantly increased in recent years, according to Table 3 analysis results. Particularly between 2023 and 2025, the number of publications increased dramatically, peaking at 12 pieces in 2025. In comparison, there were comparatively few research conducted at previous times like 2020 and 2022. This pattern suggests that AI research in Indonesian education is developing at an exponential rate, in keeping with the increased focus on digital transformation and the use of technology in education around the world.

Figure 3 illustrates the geographical distribution of the articles reviewed in this study across various regions of Indonesia. Most articles originated from Java, totalling 21, and were published in major cities such as Jakarta, Yogyakarta, Surabaya, Bandung, and Malang, indicating that Java remains a central hub for research and publication activities. Four articles from Manado, Makassar, and West Sulawesi were from Sulawesi, while four from Padang, Bukittinggi, and other areas came from Sumatra. Java contributed 21 articles in total, whereas Kalimantan, Papua, and Bali each produced three, showing a limited representation from these regions. Additionally, two articles were of multi-location or national scope. This distribution highlights a high concentration of research activity in Java, but studies from other parts of Indonesia also contribute to a diverse geographic landscape of the research analyzed.

**Figure 3.**
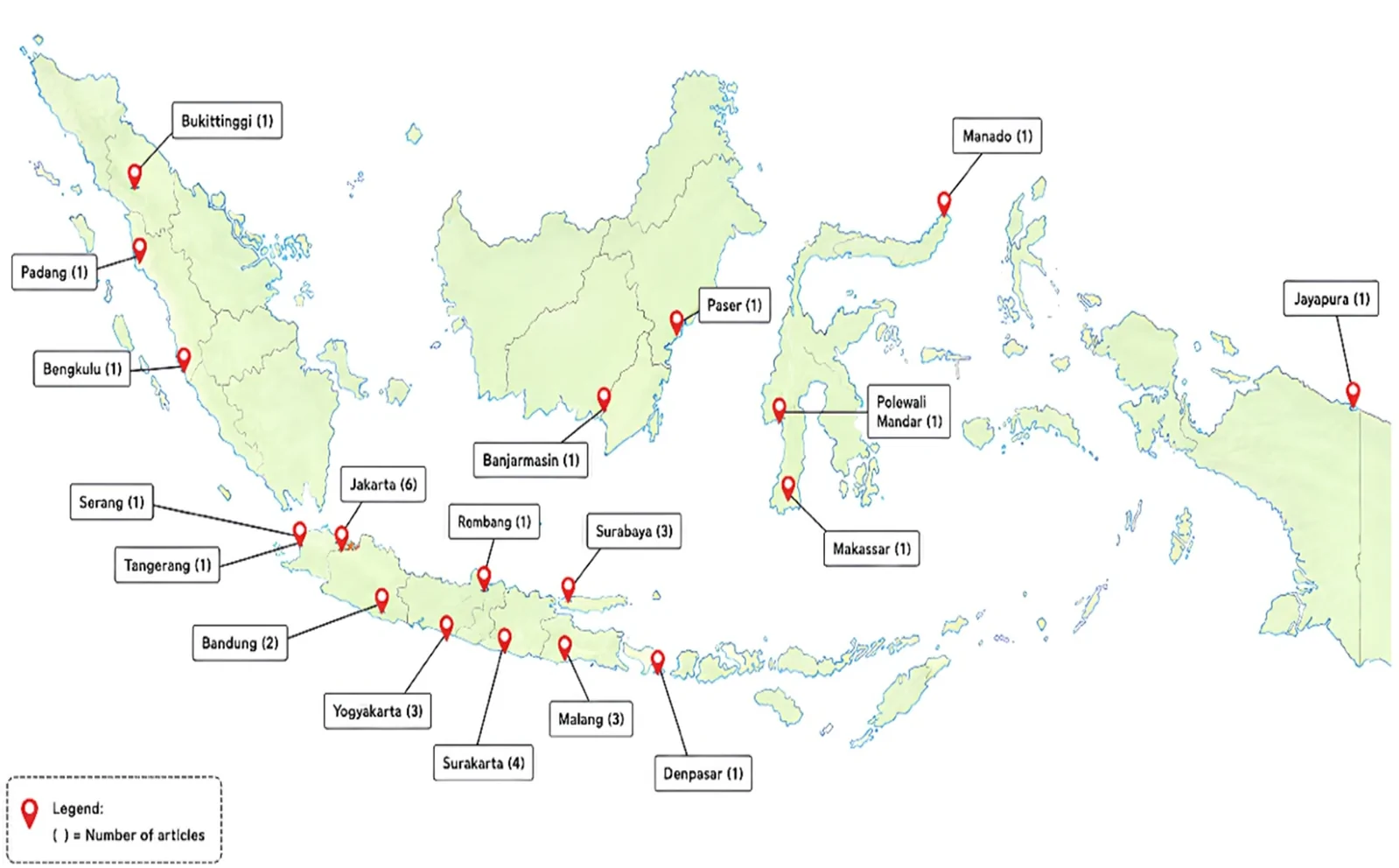
Distribution Of Articles by Location.

Language learning covering English, Indonesian, and Arabic—remains the primary focus of AI in education research in Indonesia. Significant attention has also been given to the acceptance and deployment of AI, using models like TAM and UTAUT. Other focus areas include learning innovation, students’ use of AI, and science and math instruction. However, topics such as education policy, educational system administration, and AI ethics are largely underexplored. This suggests that, although strategic and policy-oriented studies have yet to reach their full potential, AI research in Indonesia continues to emphasize practical aspects of learning.

In terms of educational levels, most studies are conducted at the higher education level, accounting for more than half of all examined papers. This indicates that universities are leading initiatives in AI research and application in education. Conversely, there is relatively little research at the primary, junior secondary, and senior secondary levels, revealing a gap that needs to be addressed in AI adoption within primary and secondary education through research and policy efforts, despite some cross-level studies. The most common techniques are qualitative approaches and literature reviews or systematic literature reviews (SLR). This points to AI education research in Indonesia still being exploratory and conceptual. Nonetheless, there is a growing use of quantitative methods, especially for assessing user acceptability and perceptions of AI, such as surveys employing SEM-PLS and technology adoption models like TAM and UTAUT. Yet, experimental studies directly measuring the effectiveness of learning with AI are scarce. This highlights the need for more experimental research to generate empirical evidence. Overall, trends and developments in AI research within Indonesian education are still in their early stages but have shown promising progress. The rising number of publications, the diversity of topics, and the variety of methodologies suggest an active and dynamic field. Nonetheless, gaps remain in distribution across educational levels, a scarcity of experimental studies, and limited research on ethics and policy. Future efforts should focus on fostering more comprehensive, cross-level, and evidence-based research to facilitate more effective and sustainable AI integration into Indonesia’s education system.

### RQ2: What forms, models, and strategies for implementing artificial intelligence are used in the learning process in Indonesia?

Incorporating artificial intelligence into the educational process is not just a reflection of technological adoption; it also entails choosing forms, models, and approaches that are specific to Indonesian educational contexts and demands. Educational academics and practitioners have employed a variety of strategies, such as the usage of AI-based chatbots, intelligent tutoring systems, adaptable platforms, and the incorporation of machine learning algorithms to create more individualized and successful learning experiences. The increasing efforts to use AI’s capabilities to address learning issues at many levels and situations of Indonesian education are reflected in the variety of forms and implementation tactics. In order to address RQ2, the following Table 4 summarizes the findings from the examined literature according to the type of AI technology employed, its learning application model, and the tactics used.

**Table 4.** Distribution of Articles Based on the Form, Model, and Implementation Strategy of Artificial Intelligence.

No.	AI Implementation Category	AI Technology/Tools Used	Application Pattern in Teaching & Learning	Education Level	No. of Articles
**1**	**Adaptive Learning & Personalized Learning**	NovoLearning, adaptive AI platforms, UTAUT framework, Virtual Mentor (LBA), Voice Assistant	AI adapts content and learning pace to each individual based on their profile and needs; pretest determines personalized learning pathway; automated real-time feedback; supports students with disabilities and blended learning environments	Elementary, Junior High, Senior High, University	7 articles (A1, A2, A14, A19, A20, A29, A30)
**2**	**Generative AI & Chatbot**	ChatGPT, Grammarly, Canva, Photomath, Socratic, NLP, SlidesGo, adaptive AR/VR	Used for developing learning modules, correcting writing and grammar, assisting with assignments, designing visual content, simulating conversations, and data-driven curriculum development	Senior High School, University	12 articles (A3, A10, A12, A15, A16, A17, A21, A23, A25, A26, A27, A31)
**3**	**AI for Instructional Media Development**	Gamma.APP, Lumen5, Teachable Machine, Canva AI, Google Classroom, Quizizz, Kahoot, Leonardo AI, Runway, CapCut	AI is used to produce slides, animated videos, and interactive media; speeds up content creation; increases student engagement and creativity; teachers serve as content curators and technology integrators	Elementary, Junior High, Senior High	6 articles (A6, A7, A8, A13, A32, A34)
**4**	**Learning Analytics & Automated Assessment**	MobileNetV2 (CNN), AI analytics, automated assessment tools, AR/VR, Meta-TAM, NLP search engine, ML recommendation system	AI analyzes individual student learning data; automated assessment using complex rubrics; personalized content recommendations; detects comprehension levels and participation; automates teachers’ administrative tasks	Elementary School, University	5 articles (A4, A11, A18, A28, A33)
**5**	**Intelligent Tutoring System (ITS) & Multi-modal AI**	Virtual AI tutor “Cicibot”, AI quiz generator, immersive VR/AR, NLP, image recognition, blockchain, AI decision-support	AI acts as an interactive virtual tutor; generates assessment questions automatically; provides adaptive individual guidance; offers immersive VR/AR experiences; processes natural language; supports data-driven pedagogical decision-making	Junior High, Senior High, University	4 articles (A5, A9, A22, A24)

The synthesis results in Table 4 show that the use of artificial intelligence (AI) in Indonesian education has various forms, models, and techniques that can be grouped into several main groups. AI implementation is not limited to a single technology but rather integrates multiple methodologies, including learning analytics, generative AI, adaptive learning, and intelligent tutoring systems. This indicates that the AI ecosystem in Indonesian education is growing in a multi-faceted way by utilising technology adjusted to educational levels, subject contexts and learning needs.

Adaptive learning and personalized learning is the first and the most common. Here AI is used to change content, learning pace and learning routes for individual students. Platforms such as Novo Learning (A1) and other AI-based systems can utilize pretests and data on student interaction to identify learning gaps, and then offer adaptive automated feedback. This is also supported by theoretical techniques such as UTAUT (A19, A29) which indicate that users’ adoption of AI is highly influenced by perceived utility and system quality. Adaptive learning is widely used in language learning, math and throughout the education spectrum from primary to post-secondary with the aim to provide a more personalized and effective learning experience.

The other kind of implementation that’s growing fastest is generative AI and chatbots especially with things like ChatGPT, Grammarly and Photomath. These technologies are used in a wide range of educational activities, including automatic language correction, conversation simulations, student task completion, and teacher-developed modules (A3, A10, A16, A21). The implementation tactics are often useful and are embedded directly into teaching and learning activities, including helping with draft writing, giving immediate feedback and increasing work efficiency. AI learning experiences are further enhanced by gamification and the integration of visual media (e.g. Canva and Quizzes). The third category includes learning analytics, automated assessment, and AI-generated educational materials. AI is used to make learning materials more interactive, e.g. automated presentations, animated movies and apps based on image recognition (A6, A7, A13). Learning analytics, on the other hand, is designed for deep analysis of student data to inform pedagogical decision making such as providing real time feedback and data driven assessment (A4, A11). Automated assessment is also a key strategy for improving evaluation efficiency, since AI can conduct evaluations quickly, without bias, and according to complex rubrics.

Finally, the use of AI is also progressing through intelligent tutoring systems (ITS) and multi-modal AI techniques that combine different technologies such as NLP, AR/VR, chatbots and intelligent recommendation systems. This approach enables more immersive, interactive and adaptive learning experiences. Examples are the use of virtual tutors (Cicibot), VR/AR in remote learning, and AI-based personalized guidance systems (A9, A22, A24). The implementation plan is strongly focused on the use of data to enable richer learning experiences, improve human-technology interaction, and provide personalized advice. Thus, the use of AI in Indonesia is not only technological but also pedagogical, transforming learning paradigms to be smarter, more flexible, and student-centered.

### RQ3: What is the impact of implementing artificial intelligence on the quality of learning in Indonesia?

An essential component of assessing how much the use of artificial intelligence actually raises the standard of education in Indonesia is impact measurement. Improvements in student learning outcomes, instructional effectiveness, student motivation and engagement, and teachers’ capacity to oversee technology-based learning are just a few of the ways that numerous studies have tried to quantify this influence. According to current research, the effects of implementing AI in education are not consistent and are impacted by a number of variables, including the kind of technology employed, infrastructure preparedness, user proficiency, and the institutional setting in which AI is applied. The following table summarizes the literature analysis results based on the assessed impact indicators, the direction of the findings, and their implications for the quality of education in Indonesia in order to address RQ3.

The synthesis results in Table 5 show that the application of artificial intelligence (AI) in Indonesian education often optimize learning outcomes. Significant gains in students’ language proficiency, conceptual understanding, writing skills and mathematical ability have been reported in many studies (A1, A2, A3, A16). Some research even showed quantitative gains, such as 35% gains in understanding (A11) and about 20% contributions of AI to exam performance (A12). This means AI can provide fast, accurate and data-driven feedback which leads to more effective and adaptable learning processes.

**Table 5.** The Impact of Implementing Artificial Intelligence on the Quality of Learning in Indonesia.

Impact Dimension	Learning Outcomes	Student Engagement	Personalized Learning	Teaching Efficiency
Learning Outcomes	AI integration significantly improved student scores and comprehension, with studies reporting 20–35% gains in performance compared to non-AI settings.	Learning outcomes were most improved when AI-driven personalization was paired with real-time feedback, enabling faster error correction and deeper concept mastery.	Automated assessment and instant feedback shortened the learning cycle, allowing students to correct mistakes immediately and progress more efficiently.	AI reinforced higher-order thinking and digital literacy skills that contribute to sustained long-term academic achievement.
Student Engagement	Motivation and active participation increased substantially; over 75% of students reported feeling more engaged when AI-based media was incorporated into learning.	Content tailored to individual interests and learning styles fostered deeper, more consistent engagement throughout the learning process.	Interactive tools such as gamification, Kahoot, and Quizizz saved instructional time while simultaneously boosting student enthusiasm.	Digital collaboration, creativity, and cross-platform communication grew through AI-facilitated learning activities.
Personalized Learning	Adaptive AI systems successfully adjusted content to match individual ability levels and learning needs, resulting in higher learner satisfaction.	Personalization emerged as the primary predictor of satisfaction and continued usage intention (β = 0.450; explaining 32.8% of variance).	Student data analysis algorithms enabled automatic content recommendations without requiring direct teacher intervention at every step.	AI-driven personalization fostered learner autonomy and adaptive learning competencies aligned with 21st-century skill demands.
Teaching Efficiency	Teachers reported significant reductions in time spent on grading and material preparation, allowing greater focus on meaningful pedagogical interactions.	Automated content customization by AI enabled teachers to concentrate on higher-level pedagogical strategies and student-centered engagement.	Automated assessment, NLP-based feedback, and data management improved operational efficiency by up to 52.9% according to reviewed studies.	Increased teaching efficiency supported educators in developing their own digital competencies and AI literacy as core 21st-century skills.
21st-Century Competencies	Students developed digital literacy, critical thinking, creativity, and communication skills through AI-integrated learning environments.	Personalized learning pathways facilitated the growth of learner independence and lifelong learning dispositions.	By automating routine tasks, AI freed up instructional time that could be redirected toward developing higher-order competencies.	AI served as a key catalyst for mastering the 4Cs (Critical Thinking, Creativity, Collaboration, Communication) in the Industry 4.0 era.

AI has been seen to increase student motivation, engagement, and learning interaction. The use of technologies such as chatbots, gamification and AR/VR leads to more interesting, dynamic and immersive learning experiences (A8, A9, A18). In addition, AI increases students’ trust in the learning process and encourages collaborative learning (A17, A22). Such high levels of student engagement and satisfaction indicate that AI, as well as a tool for cognitive support, acts as a catalyst for more fulfilling and pleasurable learning experiences.

AI is one of the main differentiating advantageous factors in individualized learning between technology-based learning and traditional ones. The AI allows tailoring the learning style, pace and content to the needs of each individual student (A4, A14, A24). Adaptive learning systems, intelligent tutoring and personalized recommendation systems have been shown to increase student happiness and effectiveness of learning. Additionally, this customization fosters student independence and motivates them to explore and comprehend content more profoundly and in context.

Artificial intelligence also plays a big role in the effectiveness of teaching and learning 21st-century skills. Findings across the selected studies indicate that AI can improve efficiency: assessment processes can be speeded up, administrative work can be automated and teachers can develop lesson plans faster and more systematically (A4, A10, A30). AI is also an agent of 21st-century skills such as critical thinking, creativity, collaboration, digital literacy and technology adaptability (A8, A20, A31). However, some studies also warn that critical thinking skills may deteriorate if the use of AI is not combined with adequate pedagogical measures. Thus, the use of AI in education should be purposeful and carefully managed.

### RQ4: What are the challenges, limitations, and opportunities for developing the implementation of artificial intelligence in learning in Indonesia?

Even though Indonesia’s use of AI in education is making encouraging strides, there are still a number of obstacles and constraints that need to be addressed in order to achieve full and long-lasting AI integration. These difficulties include regional differences in technology infrastructure, educators’ lack of digital proficiency, the scarcity of AI content created locally, and concerns about data protection and ethics when using AI-based systems in classrooms. However, these difficulties actually offer stakeholders significant chances to promote innovation, fortify technology-based education regulations, and create an AI ecosystem that is more inclusive and pertinent to Indonesia’s educational requirements. As shown in the following table, findings from the literature were thoroughly examined in order to address RQ4 based on the dimensions of problems, identified constraints, and future development prospects in Table 6.

**Table 6.** Challenges, limitations, and opportunities for developing the implementation of artificial intelligence in learning in Indonesia.

Dimension	Infrastructure Readiness	Teacher/Lecturer Competency	Ethics & Data Privacy	Education Policy
Infrastructure Readiness	Unequal internet access and limited devices remain the most pervasive barriers, especially in rural and remote areas (3 T regions). Technical issues slow loading, unstable connectivity, and limited AR/VR hardware are consistently reported across school and university settings.	Most institutions lack a systematic AI training programme. Teachers and lecturers are often self-taught, leading to uneven technology competency and heavy reliance on basic AI features.	Data privacy and security concerns are widespread across all education levels. Risks include algorithmic bias, over-reliance on AI-generated content, and threats to academic integrity (plagiarism, cheating).	No comprehensive national AI policy for education exists yet. Existing implementations are largely ad-hoc, driven by individual initiative rather than institutional or government mandate.
Teacher/Lecturer Competency	Digital and AI literacy among educators remains low. Many teachers have never used AI tools before and lack confidence in integrating them meaningfully into instruction.	Resistance to change and steep technology learning curves slow adoption. Professional development is irregular and lacks structured follow-up or peer-sharing mechanisms.	Uncritical adoption of AI risks cognitive offloading students and teachers may become overly dependent, reducing independent analytical reasoning and critical thinking.	Government-mandated AI training programmes for teachers (SD through PT) are absent. Competency standards for AI literacy in teaching have not yet been formally established.
Ethics & Data Privacy	Key ethical risks include: student data privacy breaches, algorithmic bias in AI-generated content, authenticity concerns in AI-assisted assessments, and depersonalization of the learning experience.	Lecturers lack clear institutional guidelines on ethical AI use. Around 50% of lecturers express concern that AI undermines academic integrity, yet formal protocols are rarely in place.	Ambiguous attribution, AI inaccuracy (reported at ~37%), and the risk of plagiarism are the most frequently cited ethical concerns. Transparent algorithmic standards are largely absent.	No specific AI data privacy regulation tailored to the education sector exists. Existing frameworks (e.g., PDPA) are insufficiently detailed for educational contexts.
Education Policy	Policy development consistently lags behind technological adoption. AI integration into national curricula (including Curriculum Merdeka) remains unsystematic and lacks accountability mechanisms.	Institutional support funding, technical infrastructure, and formal leadership is insufficient to sustain AI adoption at the classroom level.	Without clear policy, ethical risks go unmitigated. The absence of guidelines on AI in assessment, student data use, and content generation leaves institutions exposed.	Indonesia’s AI roadmap (Making Indonesia 4.0; National AI Strategy 2020–2045) needs accelerated implementation with specific, enforceable milestones for the education sector.
Development Opportunities	Barriers infrastructure gaps, low literacy, weak policy also define the most urgent areas for targeted development and innovation.	The demand for AI-competent educators creates an opportunity for systemic professional development programmes, including AI pedagogy training and inter-institutional knowledge sharing.	Growing awareness of ethical risks presents an opportunity to build transparent, locally accountable AI systems that embed Indonesian cultural values and educational principles.	Policy gaps signal the need and openness for evidence-based policy design, informed by multi-institutional research on AI adoption across diverse Indonesian educational contexts.

The uneven preparation of infrastructure is still the main obstacle to the integration of artificial intelligence (AI) in education in Indonesia, based on the synthesis results presented in Table 6. Most of the studies (A1, A4, A5, A11, A20, A32, A34) indicate an unequal distribution of technology between urban and rural areas, a gap in internet access and limited equipment. The 3 T (least developed, frontier and outermost) countries still face big challenges, but this tends to concentrate the use of AI in more digitally advanced places. The widespread use of AI in the country’s educational system is further complicated by several technological challenges, such as unstable connections, platform restrictions and implementation costs.

Digital skills and AI literacy remain significant barriers to human resource proficiency, particularly for instructors and lecturers. Many educators struggle with a steep technical learning curve (A13), are inexperienced with AI (A10, A34), and are reluctant to change (A19, A29). There is also concern that AI may undermine students’ ability to think critically, or replace teachers (A16, A28). It is also important to engage in continuous training, capacity building and the development of AI literacy to ensure that AI is used as a learning support tool and not as a replacement for the pedagogical function of instructors. AI also raises important considerations around data privacy and ethics. Several studies have raised concerns about algorithmic bias, data security, and possible violations of academic integrity, such as plagiarism and overreliance on technology (A12, A24, A25, A28). Moreover, learning might become less personal and there might be less social contact between students and teachers (A8, A22). The lack of a comprehensive ethical framework and explicit data protection norms indicate that stronger regulations are still required to guarantee responsible, equitable and transparent use of AI in Indonesian education.

However, Indonesia possesses a broad range of potential for the development of AI in education. The reviewed studies consistently reported that the use of AI can improve the quality of learning through automated assessment, building intelligent tutoring systems, personalized learning, and the integration of AR and VR technologies (A2, A9, A18, A31). In addition, there are opportunities to develop AI-based local contexts that are more relevant to Indonesian characteristics, such as language, culture, and citizenship education (A5, A13, A26). However, to fully exploit these possibilities and to promote an inclusive and lasting AI-based reform of education, comprehensive national policies, a precise implementation roadmap and cooperation between the government, educational institutions and the technology industry are required.

## Discussion

### The Rapid Growth of AI Research in Education in Indonesia is Uneven

The remarkable increase in the number of scholarly articles discussing the use of artificial intelligence (AI) in education in Indonesia is one of the most startling conclusions of this thorough literature assessment. Only four articles were found during the initial era (2020–2022), but this number rose dramatically to 14 during the 2023–2024 period and peaked at 16 during the 2025–2026 period, accounting for 47.1% of the 34 articles examined. This exponential development pattern is not just a statistical phenomena; rather, it represents a substantial change in scholarly focus toward the role of AI in transforming education ( Khatun et al., 2024; Zhong & Zhao, 2025).

This boom is directly related to the surge of popularity of generative AI, in particular ChatGPT by OpenAI, which was released publicly in late 2022. This technology has directly inspired academics, particularly in Indonesia, to study, document and evaluate the effects of AI in educational contexts, besides changing the way the general public interacts with AI. The phase of publications after 2023 can be called the “second phase” of AI adoption in Indonesian education, because the first phase was mainly conceptual and experimental. But this positive development is undergirded by an imbalance that needs to be corrected. Geographically, the island of Java is the focus of 61.8% of the studies (21 out of 34 papers) which include major cities such as Jakarta, Yogyakarta, Surabaya and Semarang. Kalimantan, Papua and Bali only contribute 8.8% of the total research output, while Sulawesi and Sumatra only contribute 11.8%. This pattern indicates that academic discussions on AI in education in Indonesia are still inclined to be very urban-biased and Java-centric. However, this publication pattern should not be interpreted as a direct representation of the actual distribution of AI implementation across Indonesia, because research activity, publication practices, indexing coverage, and access to research outlets may influence the available evidence.

This gap is even more pronounced when education levels are taken into account. The subject of higher education is addressed in 22 articles, which is 64.7% of all publications. There are many fewer studies at the primary, junior secondary and senior secondary levels. The situation demonstrates that universities are pioneering the investigation and use of AI because they have better human resources, digital infrastructure and research capabilities. Conversely, primary and secondary education levels are almost entirely lacking in the literature currently being published, especially in contexts with poor digital infrastructure.

This discrepancy is not merely an academic problem, but has serious policy implications. Although urban and higher education environments remain dominant in the research agenda, the policy suggestions below will likely be less relevant to most Indonesian schools, particularly those located in 3 T areas (underdeveloped, frontier and outermost regions). In order to broaden the scope of AI implementation studies to all regions in Indonesia, a concerted effort to promote more geographically and educationally inclusive AI research needs to be undertaken such as cooperative research incentives between the major universities and educational institutions in the regions and support from the Ministry of Education, Culture, Research and Technology.

### Generative AI and Adaptive Learning Dominate, But Implementation Is Still Superficial

The results of RQ2 show the progress of AI in education in Indonesia in several aspects which can be grouped into five major categories, namely learning analytics and automated assessment (5 articles), intelligent tutoring system (ITS) with multimodal AI approaches (4 articles), generative AI and chatbots (10 articles), adaptive learning and personalized learning (7 articles), and AI for learning media development (6 articles). This diversity highlights the large space of AI applications but also suggests tendencies that are worth investigating further. Generative AI and chatbots (particularly ChatGPT) are the most popular implementation type with more than one-third of all reviewed publications. Applications include aiding instructors in creating lesson plans (A10), helping students complete projects and essays (A17, A21), automating grammar correction (A3, A26), and designing visual content with AI (A16). While these applications have been shown to improve information accessibility and efficiency, the bulk of implementations are still at what one could call a “basic level use”, utilizing AI as a tool for daily chores and not as an integrated component of well-designed pedagogical frameworks.

Actually, AI’s actual educational potential goes well beyond just speeding up job completion (B. George & Wooden, 2023; Srinivasa et al., 2022). Learning outcomes have been demonstrated to be significantly impacted by deeply integrated adaptive learning systems, such as intelligent tutoring systems that adapt to individual learning styles. AI-driven personalization is a major predictor of satisfaction and continuing usage intention among Indonesian students, according to Study A14 ( Novianti et al., 2026). This conclusion is statistically significant, although it is still infrequently used as a reference in actual implementation.

This problem indicates the contradiction between the amount of technology at disposal and the depth of its application in the educational process. Many institutions are adopting AI as a reaction to trends, with no prior pedagogical planning about how AI should be incorporated into curriculum, evaluation and teacher-student interaction. That’s why AI is more of a performance enhancer than a transformer of the learning experience. To move past this superficial application, a paradigm shift is needed from simply “using AI” to “pedagogically integrating AI.” This means that the selection of AI tools should be guided by well-defined learning goals, consistent with the desired competences, and systematically evaluated for impact. Such an approach requires availability of technology but also the ability of educators to create relevant AI-based learning scenarios a skill that is still severely lacking among Indonesian lecturers and teachers.

### Advantages and disadvantages: AI associated benefits and emerging risks

The results of RQ3 and RQ4 together create a paradox that is the most complicated topic in the discussion of AI implementation in Indonesia: Evidence from the reviewed studies indicates that AI has been shown to significantly improve a number of aspects of learning quality, its unplanned application actually poses a serious risk to academic integrity, critical thinking abilities, and the preparedness of human resources in education.

1) **Documented Benefits**

From the perspective of potential benefits, several studies included in the review reported positive outcomes following the use of AI in educational contexts. Using a MobileNetV2-based mobile application to learn about ornamental plants improved student comprehension by up to 35%, according to Study A11 ( Setyawan & Purbohadi, 2025). Through adaptive and customized feedback, the AI-based NovoLearning platform successfully improves students’ English language proficiency, as Study A1 showed. When compared to classes without AI intervention, organized AI deployment can improve academic achievement by roughly 20–35% overall.

The evidence synthesized from the selected studies demonstrates AI has been improved student motivation and engagement in addition to academic results. A more engaging and entertaining learning environment is produced through the use of gamification technology, interactive chatbots, and media based on augmented reality (AR) and virtual reality (VR). When learning was combined with AI tools, more than 75% of students in the examined trials reported higher levels of engagement. The results of Study A22 ( Al Yakin et al., 2025) show that the virtual AI tutor “Cicibot” greatly enhanced pre-service teachers’ verbal and nonverbal expression and effectively promoted collaborative learning.

Another aspect of influence that consistently appears in all research is teaching efficiency. Significant time savings are reported by educators who utilize AI for automated assessment, content creation, and data administration; many studies show operational efficiency benefits of up to 52.9%. Theoretically, this “saved” time could be used for more pedagogically significant tasks like in-depth conversations, personalized coaching, and fostering students’ creativity.

2) **Lurking Risks**

But this story of advantages must be viewed in conjunction with a number of grave dangers that have also been noted in the literature. According to Study A12 ( Adiyono et al., 2025), the usage of ChatGPT in test situations in Indonesia has led to a worrying dependence: while it increases question-answering efficiency, it also weakens critical thinking abilities and jeopardizes academic integrity. AI has made it more difficult to enforce standards of academic honesty, according to almost 50% of lecturers surveyed in many studies; nonetheless, most institutions still lack explicit processes to handle this issue.

The literature refers to this threat to kids’ cognitive ability as “cognitive offloading,” and it is becoming a more pressing issue. Students’ capacity for autonomous reasoning may deteriorate if they grow accustomed to using AI to produce writing, respond to inquiries, and even solve logical puzzles. According to Study A28 ( Ninghardjanti et al., 2026), adopting AI without metacognitive awareness does not greatly enhance critical thinking abilities and, in the absence of intentional pedagogical intervention, may even worsen them. Beyond scholarly issues, difficulties with human resource preparedness also show up as a significant obstacle. The majority of Indonesian educators, particularly those working in elementary and secondary education and outside of Java, have never employed AI tools in an organized way for teaching. Current training initiatives are typically irregular, unsustainable, and lack standardized training curriculum. Therefore, pedagogical capability to maximize AI continues to be a significant barrier even in cases where infrastructure is available.

Algorithmic ethics and data privacy have likewise not gotten enough attention. The management of students’ personal data, potential biases in AI recommendation systems, and accountability for decisions made by algorithms are all significantly impacted by the usage of commercial AI platforms by educational institutions and students. Although the recently passed Personal Data Protection Law (UU PDP) still needs more precise implementing regulations for education, Indonesia currently lacks data privacy laws especially designed for the education sector.

3) **Towards a Responsible AI Implementation Framework**

Technology alone cannot overcome this benefit-risk conundrum. What is needed is a framework for the appropriate application of AI that coherently incorporates pedagogical, ethical, regulatory, and capacity-building aspects. At least four essential components should be present in such a framework. First, teacher certification programs and ongoing professional development incorporate AI literacy proficiency standards for educators. These criteria should cover more than just tool usage; they should also include pedagogical skills in creating AI-based learning.

Second, the Ministry of Education, Culture, Research, and Technology released official ethical guidelines for the use of AI in education. These recommendations include explicit limitations on the use of AI in assessment, attribution of AI-generated content, and safeguards for student data. Third, instructional design that prioritizes enhancement over replacement. Instead of taking the place of thought itself, AI should be positioned as a tool that develop students’ cognitive capacities by encouraging inquiry, analysis, and innovation. This necessitates a change in the way projects, assignments, and tests are created to make them resistant to “AI-enabled cheating” while still being pertinent to 21st-century skills.

Fourth, a national plan for implementing AI in the education sector. Current government initiatives like the government AI Strategy 2020–2045 and Making Indonesia 4.0 continue to be cross-sectoral and macro-level. A more operational roadmap with quantifiable goals, precise budgetary allotments, and evaluation procedures involving stakeholders at all levels from the school level to the ministerial level is required. Therefore, the dichotomy between the advantages and disadvantages of implementing AI in Indonesia is not insurmountable. Rather, it can act as a springboard for developing a more developed, inclusive, and accountable AI-based education ecosystem that prioritizes social justice and pedagogical ideals in addition to utilizing cutting-edge technology.

### Limitations

This study is subject to several methodological limitations that should be acknowledged. First, the final synthesis was based on a relatively small corpus of 34 articles, drawn exclusively from the Scopus and Google Schoolar databases and restricted to English-language, open-access publications. This selection process, while methodologically rigorous under the PRISMA protocol, the language criteria excluded articles published in languages other than English and Indonesian (54 articles were removed on language grounds alone). In addition, studies with restricted full-text access were excluded, potentially omitting relevant grey literature, institutional reports, and regionally focused studies that could have enriched the geographic and educational-level coverage of the review. The predominance of higher education studies (64.7%) and Java-based research sites (61.8%) in the included literature therefore reflects not only actual patterns in the field but also possible selection bias inherent to the database and language restrictions applied.

Second, the review relied primarily on self-reported outcomes and cross-sectional, largely descriptive or correlational study designs rather than longitudinal or experimental evidence, which constrains the strength of causal claims that can be made about AI’s impact on learning outcomes such as the frequently cited 20–35% performance gains. Many of the primary studies also varied considerably in sample size, measurement instruments, and operational definitions of constructs such as “engagement” or “learning quality,” making direct comparison and aggregation across studies inherently imprecise. As a qualitative-thematic synthesis rather than a meta-analysis, this review is further limited in its ability to statistically quantify the magnitude or consistency of effects across the reviewed literature, and its conclusions should therefore be interpreted as indicative of broad patterns rather than as precise, generalizable estimates.

### Future Directions

Building on the gaps identified in this review, future research should prioritize expanding the geographic and educational-level scope of inquiry beyond Java-based higher education institutions. Studies situated in primary and secondary schools, in regions outside Java, and particularly in underdeveloped, frontier, and outermost (3 T) areas are urgently needed to produce a more representative picture of AI adoption across Indonesia’s diverse educational landscape. Future work would also benefit from expanding language coverage beyond English and Indonesian, incorporating relevant grey literature, and searching additional scholarly databases to reduce potential selection bias associated with the language and database restrictions of the present review.

In terms of methodology, future studies should move beyond descriptive and correlational designs toward longitudinal and experimental or quasi-experimental research capable of establishing more robust causal evidence of AI’s effects on learning outcomes, critical thinking, and academic integrity over time. There is also a need for research that goes beyond documenting the presence of AI tools to examining how deep pedagogical integration rather than superficial task automation can be designed, implemented, and evaluated in Indonesian classrooms. Finally, future research should engage more directly with policy and ethics, including empirical evaluation of teacher AI-literacy training programs, data privacy frameworks, and the practical implementation of a national AI-in-education roadmap, so that evidence generated by scholars can more directly inform the regulatory and institutional frameworks called for in this review’s conclusions.

These findings support a recommendation to policy recommendations focus on strengthening digital infrastructure, enhancing educators’ competencies and readiness to integrate artificial intelligence (AI), and developing AI use guidelines that safeguard academic integrity, data protection, and ethical responsibility. Furthermore, governments and educational institutions should promote equitable access to technology and expand support for AI research and implementation across different educational levels and regions in Indonesia. These recommendations represent policy implications derived from the challenges and research patterns identified in the reviewed literature, rather than direct evidence of the effectiveness of specific policies.

## Conclusion

Through the examination of 34 carefully chosen scientific publications released between 2020 and 2026, this systematic literature review has effectively mapped the terrain of artificial intelligence (AI) deployment in education in Indonesia. The results from four research questions show a profession that is both structurally unequal and fast growing. Since the global introduction of generative AI technologies after 2022, research production has increased dramatically, with approximately half of all recognized papers concentrated in the 2025–2026 timeframe alone. Primary and secondary school levels, as well as areas outside of Java, are still notably underrepresented in both research and practical implementation, as this growth is still disproportionately centered on higher education institutions and Java-based urban centers. In addition to reflecting the structural inequalities already present in Indonesia’s educational system, this institutional and geographic mismatch runs the risk of continuing if future research objectives and state policy frameworks fail to address it.

From generative AI tools like ChatGPT and Grammarly to adaptive learning platforms, intelligent tutoring systems, learning analytics, and AI-powered instructional media, the review shows that Indonesian educators and researchers have embraced a wide range of AI technologies in terms of forms, models, and strategies of AI implementation. Generative AI and chatbot applications are the most often used of these, making up over one-third of all the papers that were examined. The research also indicates a prevalent pattern of shallow integration, where AI is mainly used for routine task acceleration rather than as a structurally embedded component of educational design, even though this implies widespread accessibility and simplicity of adoption. In the Indonesian context, AI’s deeper potential is still mainly untapped and underdeveloped, especially when it comes to data-driven instructional decision-making and personalized adaptive learning. A major change from tool-oriented adoption to competency-oriented pedagogical integration which links AI use with precisely specified learning objectives and quantifiable outcomes is necessary to close this gap.

The review also demonstrates that the application of AI can have significant positive effects on various aspects of learning quality when it is done in an organized and deliberate manner. The examined literature consistently shows improvements in student academic performance, motivation, engagement, and instructional effectiveness. As the main predictor of student satisfaction and persistent usage intention, personalized learning stands out as a particularly potent strategy. However, these advantages come with a number of significant and inadequately addressed drawbacks, such as the deterioration of students’ critical thinking abilities, the encouragement of academic dishonesty, worries about student data privacy, and the possibility of algorithmic bias. The key contradiction of AI adoption in Indonesian education is the cohabitation of these advantages and threats. This paradox requires a comprehensive pedagogical, ethical, and regulatory response and cannot be handled by technological solutions alone.

A multifaceted picture of Indonesia’s obstacles to long-term AI integration is painted by the difficulties and constraints found in RQ4. The most prevalent structural barrier is still infrastructure disparity, especially the ongoing digital divide between urban and rural areas. The lack of a nationally standardized AI competency framework for teachers, the lack of context-specific ethical guidelines for AI use in educational settings, the lack of education-sector data privacy regulations, and low AI literacy and capacity-building opportunities among educators all exacerbate this problem. However, the greatest strategic development opportunities are also defined by these same gaps. Purposeful innovation is encouraged by the growing need for educators with AI skills, the growing awareness of ethical issues, and the pressing need for evidence-based policy reform. A solid foundation for more inclusive, contextualized, and responsible AI implementation is provided by Indonesia’s sizable youth population, its growing edtech ecosystem, and the government’s current commitments to digital education through initiatives like Merdeka Mengajar and the National AI Strategy 2020–2045.

In conclusion, Indonesia’s use of AI in education is on a promising path, but in order to realize its full revolutionary potential, intentional multi-stakeholder action is needed. Future research should focus on longitudinal and experimental studies that produce more robust causal evidence of AI’s effects, broaden geographically to include understudied areas and educational levels, and delve deeper into issues of ethics, equity, and local contextual relevance. It is necessary for policymakers to convert current national strategies into practical roadmaps that include sector-specific goals, enforceable accountability systems, and long-term funding for AI literacy among educators. Instead of adopting AI on an as-needed basis, educational institutions should build institutionalized pedagogical frameworks that present AI as an augmentation tool that enhances rather than replaces students’ critical thinking, creativity, and cognitive growth. The ultimate objective is to create an AI-enabled learning ecosystem that is fair, pedagogically sound, morally sound, and truly responsive to the varied needs of more than 50 million students throughout the archipelago rather than just incorporating AI into Indonesia’s educational system.

## Data Availability

The dataset supporting the findings of this study is available in the Zenodo repository. This dataset forms the basis for the statistical analyzes reported in this study. The dataset can be accessed at: Riski, A. (2026). AI Integration in Indonesian Education: Trends, Outcomes, and Challenges - A Comprehensive Review. Zenodo. https://doi.org/10.5281/zenodo.21804623 Data are available under the terms of the Creative Commons Attribution 4.0 International license (CC-BY 4.0).
