# Supplementary material for: AI Integration in Indonesian Education: Trends, Outcomes, and Challenges - A Comprehensive Review
Source: F1000Res. 2026 Oct 26;15:1369. Originally published 2026 Aug 13. [Version 3] doi: 10.12688/f1000research.188536.3 (PMC13675629; doi:10.12688/f1000research.188536.3)
Supplement: Peer review history

# Reviewer report: Reviewer response for version 2

**DOI:** 10.5256/f1000research.211692.r528215

**Published:** 2026-10-22

**Authors:** Andrea FIORUCCI

**Affiliations:** 1 Universita del Salento, Lecce, Apulia, Italy

This manuscript has incorporated all the suggestions; therefore, in my opinion, it can be indexed without any further changes.

Are the rationale for, and objectives of, the Systematic Review clearly stated?

Partly

Is the statistical analysis and its interpretation appropriate?

Partly

If this is a Living Systematic Review, is the ‘living’ method appropriate and is the search schedule clearly defined and justified? (‘Living Systematic Review’ or a variation of this term should be included in the title.)

Yes

Are sufficient details of the methods and analysis provided to allow replication by others?

Partly

Are the conclusions drawn adequately supported by the results presented in the review?

Partly

Reviewer Expertise:

ICT, SPECIAL EDUCATION

I confirm that I have read this submission and believe that I have an appropriate level of expertise to confirm that it is of an acceptable scientific standard.

# Reviewer report: Reviewer response for version 2

**DOI:** 10.5256/f1000research.211692.r528216

**Published:** 2026-10-16

**Authors:** Mahlatse Ragolane

**Affiliations:** 1 Regent Business School Auckland Park, Johannesburg, South Africa

no further comments

Are the rationale for, and objectives of, the Systematic Review clearly stated?

Yes

Is the statistical analysis and its interpretation appropriate?

Partly

If this is a Living Systematic Review, is the ‘living’ method appropriate and is the search schedule clearly defined and justified? (‘Living Systematic Review’ or a variation of this term should be included in the title.)

Not applicable

Are sufficient details of the methods and analysis provided to allow replication by others?

Partly

Are the conclusions drawn adequately supported by the results presented in the review?

Partly

Reviewer Expertise:

Artificial Intelligence, Digital Transformation in Higher Education, Governance and Public Policy

I confirm that I have read this submission and believe that I have an appropriate level of expertise to confirm that it is of an acceptable scientific standard.

# Reviewer report: Reviewer response for version 2

**DOI:** 10.5256/f1000research.211692.r528401

**Published:** 2026-10-12

**Authors:** Muralidhar Kurni

**Affiliations:** 1 Department of Computer Science and Engineering, Anantha Lakshmi Institute of Technology & Sciences, Anantapuramu, Andhra Pradesh, India

**Reviewer Report –**

 The manuscript, “AI Integration in Indonesian Education: Trends, Outcomes, and Challenges – A Comprehensive Review,” presents a systematic literature review of 34 studies published between 2020 and 2026. It examines research trends, AI implementation approaches, educational outcomes, and challenges associated with AI adoption in Indonesia. The study highlights the growing use of AI in higher education, geographical differences in research activity, and important concerns relating to digital infrastructure, teacher preparedness, academic integrity, and ethical governance.

 The topic is timely and relevant, and the review has the potential to provide useful insights for researchers, educators, and policymakers. I appreciate the authors' efforts to improve the revised manuscript, particularly the clearer organization around four research questions and the additional discussion of methodological procedures and study limitations.

 However, several issues remain that should be addressed to improve the scientific reliability and reproducibility of the review.

**1. Rationale and objectives – Yes**

 The rationale and objectives are clearly stated. The four research questions provide an appropriate framework for examining AI integration in Indonesian education. However, the authors could strengthen the introduction by explaining more explicitly how this review contributes beyond existing literature reviews on the same topic.

**2. Methods and reproducibility – Partly**

 The authors have provided useful information about the literature selection process and thematic analysis. Nevertheless, some important inconsistencies remain.

- **Essential:** The databases used for the literature search should be reported consistently. The methodology mentions Scopus and Crossref, while the abstract, PRISMA diagram, and Table 1 refer to Scopus and Google Scholar.
- **Essential:** The publication period must be clarified. Table 1 specifies 2015–2025, whereas other sections indicate 2020–2026.
- **Essential:** The language eligibility criteria should be clarified, particularly regarding the inclusion or exclusion of Indonesian-language publications.
- **Essential:** The authors should provide sufficient information about the search dates, Google Scholar search procedure, study selection, and quality appraisal to allow other researchers to reproduce the review.
- **Essential:** The exclusion of articles without citations and those with restricted full-text access requires justification, as these criteria may introduce selection bias.

**3. Statistical analysis and interpretation – Partly**

 The descriptive and thematic approach is appropriate for the objectives of the review. However, the numerical findings require further verification.

- **Essential:** The publication counts in Table 3 do not reconcile with the stated total of 34 studies. These values should be corrected and checked against Figure 2 and the corresponding discussion.
- **Essential:** The authors should verify the consistency of all reported counts, percentages, and classifications across the tables, figures, and text.
- **Essential:** Reported improvements in learning performance, including the 20–35% figures, should be clearly attributed to individual studies rather than presented as generalizable effects of AI integration.
- The discussion should distinguish more clearly between experimental evidence, survey findings, and descriptive observations.

**4. Conclusions and supporting evidence – Partly**

 The conclusions are relevant and generally reflect the major themes identified in the review. However, some statements should be interpreted more cautiously.

- **Essential:** Conclusions about improvements in learning outcomes and teaching efficiency should reflect the methodological limitations of the included studies.
- **Essential:** The concentration of reviewed publications in Java and higher education should not automatically be interpreted as evidence of the actual distribution of AI implementation throughout Indonesia.
- The policy recommendations are valuable, but the authors should distinguish findings directly supported by the reviewed literature from their broader recommendations for future educational development.

**5. Living systematic review – Not applicable**

 The manuscript is not presented as a living systematic review. Therefore, the assessment of continuous updating procedures and a predefined search schedule is not applicable.

**Overall Assessment**

 The revised manuscript addresses an important research area and offers a useful overview of AI integration in Indonesian education. The authors have made meaningful improvements in response to earlier reviewer feedback.

 Nevertheless, the remaining methodological inconsistencies, numerical discrepancies, and limitations in interpreting the evidence need further attention. The points identified as essential should be addressed to ensure the accuracy, transparency, and scientific soundness of the review.

 I encourage the authors to undertake a careful final verification of the methodology, tables, figures, and conclusions. Addressing these concerns would considerably strengthen the manuscript and improve its value to the research community.

**Recommendation: Approved with Reservations.**

Are the rationale for, and objectives of, the Systematic Review clearly stated?

Yes

Is the statistical analysis and its interpretation appropriate?

Partly

If this is a Living Systematic Review, is the ‘living’ method appropriate and is the search schedule clearly defined and justified? (‘Living Systematic Review’ or a variation of this term should be included in the title.)

Not applicable

Are sufficient details of the methods and analysis provided to allow replication by others?

Partly

Are the conclusions drawn adequately supported by the results presented in the review?

Partly

Reviewer Expertise:

Artificial Intelligence, Machine Learning, AI in Education, Educational Technology, Learning Analytics, and Generative AI

I confirm that I have read this submission and believe that I have an appropriate level of expertise to confirm that it is of an acceptable scientific standard, however I have significant reservations, as outlined above.

# Response

**Published:** 2026-10-21

**Authors:** Novebri Novebri

**Affiliations:** Educational Administration, Universitas Pendidikan Indonesia, Bandung, West Java, Indonesia

We sincerely thank the reviewer for the constructive and valuable feedback. We have carefully revised the manuscript in accordance with the suggestions, particularly by clarifying the research contribution, improving the consistency of the methodology, verifying the numerical findings across tables and figures, and refining the interpretation of the results and policy recommendations to ensure they remain aligned with the available evidence. We would also like to note that several concerns raised in Points 2 and 3 overlap with comments previously provided by Reviewers 1 and 2, which we have already addressed in the previous revision. Nevertheless, we have re-examined these aspects to ensure consistency, accuracy, and completeness throughout the manuscript. We believe that these revisions have improved the transparency, reproducibility, and scientific rigor of the review. We appreciate the reviewer’s thoughtful recommendations, which have helped us further strengthen the quality and clarity of our manuscript.

# Reviewer report: Reviewer response for version 1

**DOI:** 10.5256/f1000research.208079.r520173

**Published:** 2026-09-26

**Authors:** Andrea FIORUCCI

**Affiliations:** 1 Universita del Salento, Lecce, Apulia, Italy

The abstract cites Scopus and Sinta and a 2020–2026 timeframe. The methodology section, however, mentions Scopus and Crossref and articles published between 2015 and 2025. Yet, in the PRISMA diagram, the 773 records originate from Sinta and Scopus again.

 Table 1 appears to contain extraneous material from another study.

 The “Search Keyword” entry lists “Women leadership, female leadership, women leaders, gender and leadership…”, which is clearly irrelevant to a review on AI in education. Furthermore, the same table lists “Scopus and crossreff” and subsequently Google Scholar, whereas the text specifies Sinta.

 The text states that articles could be in English or Indonesian, yet the table lists “English” as the sole language criterion, and the PRISMA diagram notes the exclusion of 54 “non-English” papers. No critical appraisal tool is described, nor is there an explanation of how the methodological quality of the 34 selected contributions influenced the synthesis. This is a significant limitation for a systematic review. It is unclear how many reviewers independently screened titles/abstracts and full texts, how disagreements were resolved, which data extraction form was used, which variables were extracted, and how thematic categories were constructed. The Introduction poses five main questions, including system readiness and ethical-legal aspects. The methodology section, however, presents four research questions (RQs). I suggest adopting the four RQs consistently and realigning the Introduction, methodology, coding, and results.

 There are significant numerical inconsistencies regarding the time trends. Table 3 lists 4 articles for 2020–2022, 14 for 2023–2024, and 16 for 2025–2026; however, Figure 2 and the accompanying text present a different annual distribution and indicate different peaks. The text correctly identifies 21 studies from Java but subsequently states that “Java contributed eight articles in total”—a clear contradiction of the previously reported figures and of Table 3, which attributes 61.8% of the articles to Java. Regarding “Generative AI & Chatbot,” the text cites “10 articles,” yet lists twelve codes: A3, A10, A12, A15, A16, A17, A21, A23, A25, A26, A27, and A31. Claims such as “20–35% gains in performance” or efficiency improvements of up to 52.9% are presented almost as aggregate estimates of AI’s impact; however, no meta-analysis was conducted, and the studies are heterogeneous in terms of design, population, tools, and outcomes. The Discussion introduces interpretive categories that lack a sufficient basis in the analytical procedure.

 Terms such as “second wave,” “surface level use,” and “benefit-risk paradox” offer interesting interpretations, but it is unclear how they were derived from the systematic data coding. They may be retained, provided they are clearly presented as the authors’ interpretations rather than categories that emerged directly from the review.

 The conclusions should be framed more cautiously.

Are the rationale for, and objectives of, the Systematic Review clearly stated?

Partly

Is the statistical analysis and its interpretation appropriate?

Partly

If this is a Living Systematic Review, is the ‘living’ method appropriate and is the search schedule clearly defined and justified? (‘Living Systematic Review’ or a variation of this term should be included in the title.)

Yes

Are sufficient details of the methods and analysis provided to allow replication by others?

Partly

Are the conclusions drawn adequately supported by the results presented in the review?

Partly

Reviewer Expertise:

ICT, SPECIAL EDUCATION

I confirm that I have read this submission and believe that I have an appropriate level of expertise to confirm that it is of an acceptable scientific standard, however I have significant reservations, as outlined above.

# Response

**Published:** 2026-09-29

**Authors:** Novebri Novebri

**Affiliations:** Educational Administration, Universitas Pendidikan Indonesia, Bandung, West Java, Indonesia

Thank you very much for these valuable and constructive comments. We have carefully revised the manuscript to address all concerns raised. The databases, publication timeframe, language criteria, and eligibility criteria have been harmonized across the Abstract, Introduction, Methodology, PRISMA diagram, and tables. Table 1 has been corrected by removing irrelevant material and updating the search keywords and methodological information to reflect the present AI-in-education review. We have also strengthened the methodological description by clarifying the critical appraisal process, reviewer involvement, disagreement resolution, data extraction procedures, extracted variables, and thematic coding process. The Introduction and Methodology have been aligned to consistently use the four research questions (RQs).

 All numerical inconsistencies have been rechecked and corrected. In particular, the number of articles in Table 3 has been revised and revalidated to ensure consistency with Figure 2 and the corresponding narrative, including publication periods and geographical distribution. The inconsistencies concerning studies from Java and the “Generative AI & Chatbot” category have also been corrected. Quantitative findings such as the reported 20–35% learning-performance improvement and 52.9% efficiency improvement are now explicitly attributed to the respective individual studies and are clearly presented as study-specific findings rather than pooled estimates. We have also distinguished associative, self-reported, and experimental evidence and moderated causal language accordingly.

 Finally, the terms “second wave,” “surface-level use,” and “benefit–risk paradox” have been retained but explicitly framed as the authors’ interpretations of recurring patterns rather than categories directly generated through coding. The Discussion and Conclusion have also been revised to use more cautious, evidence-based language. We sincerely appreciate the reviewer’s guidance, which has improved the manuscript’s methodological consistency, analytical transparency, numerical accuracy, and interpretive rigor.

# Reviewer report: Reviewer response for version 1

**DOI:** 10.5256/f1000research.208079.r520172

**Published:** 2026-09-12

**Authors:** Mahlatse Ragolane

**Affiliations:** 1 Regent Business School Auckland Park, Johannesburg, South Africa

The rationale, research questions and objectives are explicitly stated and well aligned with the topic.  The concern is methodological reproducibility. The manuscript contains inconsistencies regarding databases and dates for example the abstract reports Scopus/Sinta and 2020-2026, whereas the methods/table describe Scopus/Crossref and 2015-2025, with Google Scholar also appearing in the search table. The review also does not adequately describe study-quality/risk-of-bias assessment, reviewer procedures, data-extraction methods, or how disagreements were resolved. These issues must be addressed to make the review scientifically sound. The thematic synthesis is reasonable for heterogeneous literature, but quantitative claims such as 20-35% performance gains and 52.9% efficiency improvements require clearer study-level attribution and methodological qualification. The authors themselves acknowledge substantial heterogeneity and the absence of meta-analysis. The conclusions are directionally consistent with the review but sometimes stronger than the underlying evidence warrants, particularly causal claims about AI improving learning. The authors should distinguish association, self-reported outcomes and experimental evidence.

Are the rationale for, and objectives of, the Systematic Review clearly stated?

Yes

Is the statistical analysis and its interpretation appropriate?

Partly

If this is a Living Systematic Review, is the ‘living’ method appropriate and is the search schedule clearly defined and justified? (‘Living Systematic Review’ or a variation of this term should be included in the title.)

Not applicable

Are sufficient details of the methods and analysis provided to allow replication by others?

Partly

Are the conclusions drawn adequately supported by the results presented in the review?

Partly

Reviewer Expertise:

Artificial Intelligence, Digital Transformation in Higher Education, Governance and Public Policy

I confirm that I have read this submission and believe that I have an appropriate level of expertise to confirm that it is of an acceptable scientific standard, however I have significant reservations, as outlined above.

# Response

**Published:** 2026-09-29

**Authors:** Novebri Novebri

**Affiliations:** Educational Administration, Universitas Pendidikan Indonesia, Bandung, West Java, Indonesia

Thank you for this valuable comment. We have carefully revised the relevant sections of the manuscript in accordance with the reviewer’s suggestions. Specifically, the reported quantitative findings, including the 20–35% improvement in learning performance and the 52.9% efficiency improvement, have been explicitly attributed to the respective individual studies from which these findings were derived. We have also clarified that these figures represent study-specific findings rather than pooled estimates from a meta-analysis. In addition, the discussion and conclusion have been revised to distinguish between associative findings, self-reported outcomes, and experimental evidence. Causal language has also been moderated where necessary to ensure that the conclusions remain proportionate to the underlying evidence and the methodological characteristics of the reviewed studies.
